# A molecular analysis by gene expression profiling reveals *Bik/NBK *overexpression in sporadic breast tumor samples of Mexican females

**DOI:** 10.1186/1471-2407-5-93

**Published:** 2005-08-01

**Authors:** Normand García, Fabio Salamanca, Horacio Astudillo-de la Vega, Everardo Curiel-Quesada, Isabel Alvarado, Rosenda Peñaloza, Diego Arenas

**Affiliations:** 1 Molecular Genetics Laboratory, Medical Research Unit (MRU), Pediatric Hospital, National Medical Center Century XXI, Mexican Social Security Institute, Mexico City, Mexico; 2 Molecular Oncology Laboratory, Oncology Hospital, National Medical Center Century XXI, Mexican Social Security Institute, Mexico City, Mexico; 3 Department of Anatomy-Pathology, Oncology Hospital, National Medical Center Century XXI, Mexican Social Security Institute, Mexico City, Mexico; 4 Genetics Engineering Laboratory, Biochemical Department, Biological Sciences National School, Polytechnic National Institute. Mexico City, Mexico

## Abstract

**Background:**

Breast cancer is one of the most frequent causes of death in Mexican women over 35 years of age. At molecular level, changes in many genetic networks have been reported as associated with this neoplasia. To analyze these changes, we determined gene expression profiles of tumors from Mexican women with breast cancer at different stages and compared these with those of normal breast tissue samples.

**Methods:**

^32^P-radiolabeled cDNA was synthesized by reverse transcription of mRNA from fresh sporadic breast tumor biopsies, as well as normal breast tissue. cDNA probes were hybridized to microarrays and expression levels registered using a phosphorimager. Expression levels of some genes were validated by real time RT-PCR and immunohistochemical assays.

**Results:**

We identified two subgroups of tumors according to their expression profiles, probably related with cancer progression. Ten genes, unexpressed in normal tissue, were turned on in some tumors. We found consistent high expression of *Bik *gene in 14/15 tumors with predominant cytoplasmic distribution.

**Conclusion:**

Recently, the product of the *Bik *gene has been associated with tumoral reversion in different neoplasic cell lines, and was proposed as therapy to induce apoptosis in cancers, including breast tumors. Even though a relationship among genes, for example those from a particular pathway, can be observed through microarrays, this relationship might not be sufficient to assign a definitive role to *Bik *in development and progression of the neoplasia. The findings herein reported deserve further investigation.

## Background

Breast cancer is one of the most frequent causes in Mexican women over 35 years of age and mortality shows a tendency to increase over time [1]. The origin and course of sporadic breast cancer are not clear. At the molecular level, some alterations have been reported as associated with this neoplasia, such as changes in DNA quantity [2], cytogenetic alterations [3], amplification of some proto-oncogenes [4,5], loss of heterozygosity in some chromosomal regions [6,7], and mutations in at least four different susceptibility genes in hereditary forms [8-11].

The traditional way of classifying breast tumors is based on tumor size, degree of dissemination and Histopathology. Alterations in many genetic networks are involved in development of breast cancer; therefore, analysis of isolated genes is not sufficient to adequately understand this neoplasia. Transcriptional analysis of multiple genes expressed by breast tumors should provide a means to define a signature or molecular fingerprint of the disease and might progressively replace conventional diagnostic and prognostic parameters. Likewise, the capability to analyze simultaneous expression levels of thousands of genes offers better possibilities to understand and characterize the complete molecular mechanisms underlying cancer progression. Technologies such as DNA chips permit integral study of the advance of the disease, with the advantage of identifying marker genes for diagnosis, prognosis, and therapy.

In several studies found correlations among expression profiles and clinical characteristics, estrogen receptors, lymphatic nodes or treatment response [12-16]. However, this results can not be generalized to other populations because development of this heterogeneous disease have influence from multiple factors, including age, diet, genetics, environment, geographic location, no pregnancy and race [17]. To generate portraits for each population may help in the sub-classification of tumours, prognosis, and general understanding of breast cancer [18,19] and will allow us to identify characteristic genes from the Mexican population.

Tumor growth rates can be <5% of those predicted by proliferation measurements alone. Several types of human cancers such as colorectal [20], ovarian [21], endometrial [22], and cervical [23], also showed an increase in apoptotic index (by TUNEL assay) during tumorigenesis, which questions if cancer research fields related with inhibition of cell death might be a critical step in cancer development. Thus, in these cases a high rate of cellular proliferation must be responsible for tumor growth.

Human gene expression patterns derived from cDNA microarrays have been increasingly used to identify genes associated with human cancers [12,14,24]. On the basis of these studies, it appears that cDNA microarray based gene expression analysis of breast cancers tissues would reveal molecular characteristics associated with tumorigenesis.

Bcl-2 family proteins contain both, anti-apoptotic and pro-apoptotic, members and are essential to maintain organ systems. For many, but not for all apoptotic signals, balance between these two Bcl-2 subfamilies determine cell fate. The pro-apoptotic Bik protein is a member of one sub-class of the Bcl-2 family designated BH3-alone [25]. The human *Bik *gene is located on 22q13.3 and codifies a 160 amino acid protein. Its mRNA has ubiquitous distribution with elevated levels in heart and skeletal muscle [26].

Recently, the product of *Bik *gene has been associated with tumoral reversion in different cell lines and was proposed as therapy to induce apoptosis in cancer including breast tumors [27,28]. Using cDNA microarrays, we obtained gene expression profiles of 15 breast cancers and 5 normal breast tissues. Complete pairwise comparison of selected genes with *real-time *RT-PCR revealed consistent overexpression of *Bik/NBK *gene in tumor samples.

## Methods

### Breast tissue samples

The protocol of this work was approved by the Ethical Committee of our hospital with register number 98/718/43 and all patients provided informed consent in signed letter prior to the initiation of any procedure. Samples were taken from non-affected breast tissue and affected tissue from 15 non-related Mexican patients 40 years of age [mean of 52 years and range 40–68 years]. All tumors were sporadic, infiltrating, ductal adenocarcinomas from patients who had not received adjuvant chemotherapy. Tissues were obtained from the Oncology Hospital, at the Centro Medico Nacional Siglo XXI of the Instituto Mexicano del Seguro Social in Mexico City. Each sample was snap-frozen in liquid nitrogen and stored at -70°C until use. Specimens were selected for analysis by two criteria: (i) sufficient material for analysis, and (ii) histological evaluation by two pathologists demonstrating that samples contained at least 70% of tumor cells (in the case of cancer samples). Representative sections from each case were paraffin-embedded for staining with haematoxylin and eosin to assess histopathologic diagnosis using American Cancer Committee criteria [29] and for their use in Immunohistochemistry assays.

### RNA extraction and mRNA purification

Total RNA of each sample was obtained with trizol and dissolved in *RNase-free *water for a final concentration of 1–2 μg/μl. Samples was stored at -70°C. Concentration and purity of each sample was assessed by gel electrophoresis and spectrophotometry (Ultrospec 2000, Pharmacia Biotech). Prior to obtaining poly A+ RNA, total RNA was treated with DNAse I according to Atlas pure total RNA labeling system (Clontech, Palo Alto, CA, USA). Poly A enrichment procedure was used to obtain mRNA (Atlas pure total RNA labeling system^®^, Clontech, Palo Alto, CA, USA).

### Probe labeling and hybridization

cDNA label procedure was used to obtain ^32^P-labeled cDNA from 1 μg of poly A, with MoMLV reverse transcriptase (Atlas pure total RNA labeling system^®^, Clontech, Palo Alto, CA, USA). Labeled probes were purified with chroma SPIN-200 columns (Atlas cDNA Expression Arrays^®^, Clontech, Palo Alto, CA, USA). Incorporation of ^32^P into the probe was double-checked in a scintillation counter (Beckman, model LS 9000sc).

The hybridization procedure was performed with hybridization solution mixed with the entire pool of labeled cDNA probes with > 1.25 × 10^6 ^cpm on Atlas array membrane with 609 genes, double-spotted (Atlas human cancer cDNA expression arrays^®^; Clontech, Palo Alto, CA, USA). The membrane was hybridized at 68°C for 24 h, and then was washed with high-to-low astringency solutions. Membranes were exposed for 24 h in a phosphor screen (Kodak storage phosphor screen, Molecular Dynamics).

### Image analysis and data collection

The digitalized image was obtained in a storm scanner (Storm 680, Molecular Dynamics, Inc.). Expression profiles were obtained with Atlas image 2.0 software (Clontech, Palo Alto, CA, USA) through comparison of normal *vs*. tumor tissues. We used a normalization coefficient in which average value of all genes was used to normalize the array. This coefficient was determined with the Sum method, which adds values of signal over background for all genes on arrays.

### Data analysis

Hybridization profiles were analyzed with J-Express program developed and distributed by Molmine AS and collaborators [30], and Cluster and TreeView software's [31].

### Real Time-RT-PCR analysis

The same source of total RNA used to define gene expression profiles was used in real time RT-PCR experiments, following all instructions outlined in the LightCycler-RNA amplification kit SYBR Green I manual (Roche Molecular Biochemicals, Mannheim, Germany). cDNA synthesis was carried out in LightCycler (Roche) in a capillary as follows: 20 μl mix reaction containing 500 ng of DNase I treated total RNA, 4 μl of LightCycler-RT-PCR reaction mix SYBR Green I (final concentration 1X), 5 mM MgCl_2_, 0.4 μl LightCycler-RT-PCR enzyme mix, and 5.0 pmol forward and reverse primers for both genes. *Bik *gene primer sequences were designed with OLIGO 4.1 and were as follows: forward 5' GAG ACA TCT TGA TGG AGA CC 3', reverse 5' TCT AAG AAC ATC CCT GAT GT 3'. *HPRT *gene primers were referred by Pieretti M *et al; *1991 [32]. For reverse transcription, the reaction was incubated at 55°C for 30 min and at 95°C for 30 sec.

Amplification was carried out in the same capillary. LightCycler was programmed as follows: 50 three-segment cycles for amplification (10 sec at 95°C, 30 sec at 55°C, and acquisition (single-mode), 20 sec at 72°C) and three-segment cycle of product melting (0 sec at 95°C, 10 sec at 65°C, and 0 sec at 95°C at step-acquisition mode). Temperature transition rate for all segments of amplification cycles and melting curve cycle were set at 20°C/sec except for segment 3 that was set at 0.1°C/sec of product-melting curve analysis. Duplicate reactions were prepared for each sample along with a non-template negative control (H_2_O control). A standard curve was used with these assays and LightCycler3 data analysis software (Roche Molecular Biochemicals) was used in all processes.

### Immunohistochemistry

For identification of Bik (NBK) protein expression, streptavidin-biotin peroxidase complex (DAKO LSAB Kit, Carpinteria, CA, USA) method with diaminobenzidine as chromogen was used. Epitopes were retrieved by autoclaving in 10 mM citric acid buffer, pH 6.1 for 2 min. As primary antibody, polyclonal anti-human NBK/Bik FL-160 (Santa Cruz Biotechnology, work dilution 1:100) in 1% bovine serum albumin-phosphate buffered saline (BSA-PBS) was used. Formalin-fixed, paraffin-embedded sections of normal skin epithelium served as positive controls for Bik (NBK). Negative control slides were processed in parallel, omitting primary antibody.

## Results

### Expression profiles of normal and malignant breast specimens

We analyzed gene expression patterns in dissected normal or malignant human breast tissue from 20 individuals, 15 infiltrating ductal carcinomas, nine stage II, six stage III, and five normal breast samples. Hierarchical cluster analysis was used to group genes on the basis of similarity in their patterns of expression. We used average cluster linkage, uncentered [31] based on *Euclidean *distance and removed all genes that were turned on in tumors but that were not expressed in normal tissue (*NCK5AI, CDK5 activator, CDC25A, ERK4, K2P, COL11A1, OBCAM, AMPHIREGULIN, BCGF1 *and *BMP8*).

**Figure 1:**
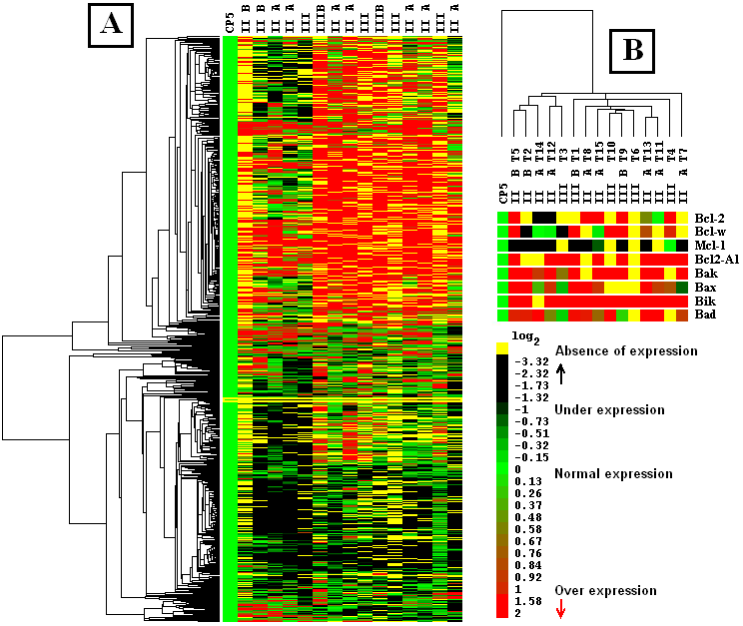
**Dendrogram and picture of hierarchical cluster of 15 mammary tumors and normal tissue**. Expression profiles from tumor and normal tissue (average from five normal tissues) were obtained through average-linkage, hierarchical, uncentered clustering analysis, as described in Methods. Genes unexpressed in normal tissue were not included in analysis. In dendrograms, each branch corresponds to similar expression profile among genes (A) or samples (B). Green squares in picture correspond to normal expression levels. Yellow squares correspond to absence of expression. Black squares correspond to underexpressed genes. Red squares correspond to overexpressed genes. Picture under dendrogram in panel details behavior of eight apoptosis-related genes along samples.

Cluster analysis of tumors and controls is described in Figure 1. Patterns of gene expression among tumors showed great variation (Figure 1A); however, we identified two main groups. The left branch is conformed by four histologic type II and one type III, while the right branch contains five type II and five type III. Left branch shows distinctive underexpression and loss of expression areas with genes such as *IFNGR2, interleukin 2, laminin B1, MSH2, MSH6 CASP8 and 10, RB1, PCNA, EGFR *and *PGS2*, among others, and a few overexpressed genes such as *MIF, CDK 2, 5*, and *7, RPSA, JUP *and *ITGAE*. On the other hand, right branch shows overexpression areas with genes such as *CASP 10, CD30-L*, and *CD-40-L *(TNF family), *ras-like small, FGF 3 and 5, TDGF1, ERK5, MIF, IFNGR2*, *MIG, IFN-alpha, beta, gamma, IL-3, -4, -9 *and *-13, IP-10*, *HPRT, DCC, TEK, netrin-2, cadherins 4, 8, 12 *and *13 *[33], *MMP3, 8, 10, 12 *and *13 *(metalloproteinase), *COL11A2, CCNG1, CDC-6, ATM, N-MYC*, and *p53*.

In Figure 1B, tumors and controls were associated in a dendrogram obtained with expression profile of each sample. With this analysis, tumors were separated in two main branches; left group contained five tumors defined predominantly by histologic type II, while right group was composed by stage II and III tumors, at the same percentage. In the lower part of the same Figure, we showed the gene expression pattern of eight genes related with apoptotic process obtained from each tumor, showing changes across all samples but exposing the possible relation between pro-apoptotic (*BAK, BAX, BIK, BAD*) and anti-apoptotic genes (*BCL2, BCLW, MCL1, BCL2- A1*). We showed that pro-apoptotic BCL2-interacting killer gene (*BIK/NBK*) overexpressed in nearly all samples (14/15), which makes it a possible candidate for further studies on its role in breast cancer. *MCL1 *was underexpressed or missing, at least 93.3% (14/15), relative to controls.

Due to consistent high expression of *Bik *gene in breast cancer, we validated its overexpression by real-time PCR and studied distribution of Bik protein in tumor breast samples by Immunohistochemistry.

### Real time RT-PCR assay for Bik gene

**Figure 2:**
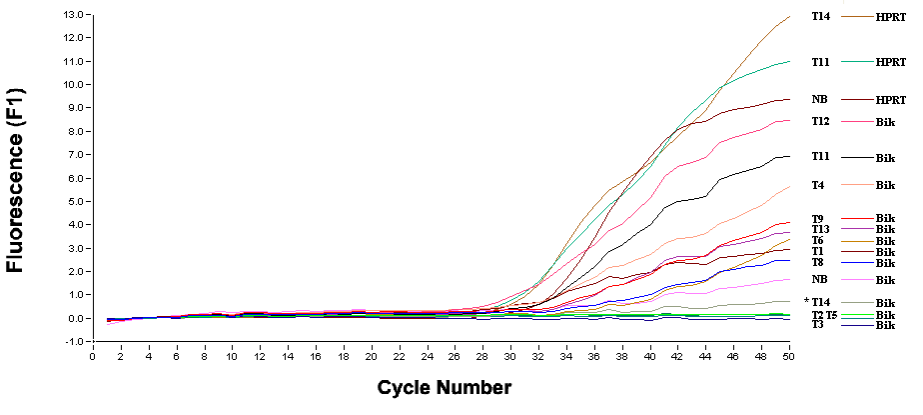
**Real time RT-PCR validation of expression *Bik *and *HPRT *genes**. Amplification curves of *Bik *(Bik T1 to T14), *HPRT *(T11 and T14) and non-affected breast (NB) mRNAs are shown. Graphic generated by LightCycler-based real time PCR thermocycler. The abscissa shows fluorescence relative units and ordinate cycle number.

According to the Methods section, we developed real-time RT-PCR using *Bik *and *HPRT *mRNAs as templates. In Figure 2, we showed amplification products of different samples of *Bik *and *HPRT *genes. With real-time RT-PCR, results of 9/13 samples (69%) agreed with those obtained through microarray analysis (considering NB and T14) and in 31% of tumors, we were unable to confirm the result. By using melting curves (data not shown), we confirmed that amplification products obtained with real-time RT-PCR were specific. As positive control, *HPRT *gene (housekeeping) was used (samples numbers 11 and 14) for tumors and normal breast (NB). We used tumor 14 as negative control (tumor that wich show expression for *Bik *gene in microarray assay).

### Characterization of Bik/NBK expression by immunohistochemistry

**Figure 3:**
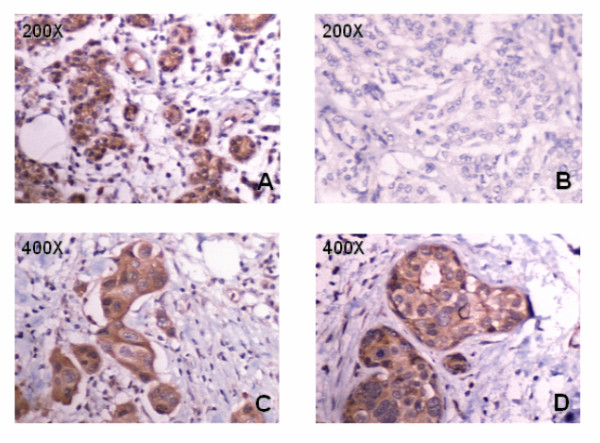
**Distribution of Bik protein in mammary ducts from adenocarcinomas**. Anti-Bik/NBK polyclonal antibody) was reacted with HRP-secondary antibody and revealed with DAB. Haematoxylin was used as counter stain. A, C, and D are different tumor areas. B, negative control from the same patient.

After RT-PCR analysis, we checked the distribution and abundance of Bik protein on slides obtained from the same tissue used for histopathologic diagnosis. Only in poorly or well-differentiated tumor areas we found presence of BIK protein with predominant cytoplasmic location (Figure 3A). Figures 3C and 3D (magnification to 400×) show Bik protein in a sample of breast cancer. Presence of BIK protein was specific because, when primary antibody was omitted, we did not observe any signal in neoplasic tissue (Figure 3B).

## Discussion

Advances in high-density DNA microarray technology have permitted to screen large numbers of genes and to correlate tumoral stages and gene-expression profiles in cancer research. The idea is that tumor behavior is ruled by expression of hundreds of genes; thus microarrays analysis allows those behaviors and clinical features to be predicted. In the present study, 10 genes were turned on in some tumors respect to the normal tissue.

With microarray analysis of samples, we identified two subgroups probably related with cancer progression. In the first group, we identified an underexpression area (lower part of dendrogram in Figure 1) Wich corresponded to four histological stage II tumors, (T2, T5, T12 and T14) and one tumor in histological stage III, (T3). The latter tumor probably corresponded to a stage II tumor at molecular level because its profile expression was similar to others in the same group. We think that this group belongs to an initial stage of development of neoplasia, because it presents fewer alterations than the second group, and because the behavior of some genes related with the beginning of tumor development such as genes related with DNA repairment, apoptosis, TNF, Fas-L route, checkpoint, remodeling, and maintenance of extracellular matrix, were underexpressed or lost their expression while other genes were overexpressed, such as human macrophage migration inhibitory factor and some cyclin-dependent kinases and cytoskeletal proteins.

The right branch (Figure 1) contains stage II or III tumors and probably correspond to more advanced stages of progression of neoplasia. In this group, we found some overexpressed genes; of these, *COL11A2 *and *netrin-2 *had not been previously associated with breast cancer. Other genes, such as *DCC *and *cadherin 13*, were found overexpressed in our samples but underexpressed in other reports [34]; these genes probably have mutations that differentially affect their proteins.

Apoptosis is a mechanism to control cell death which occurs during normal development, growth, and maintenance in multicellular organisms. It can be activated by stimulation of a cell surface death receptor or release of cytochrome c from mitochondria. In the cascade of events initiated by this signaling, cysteine aspartyl proteases, or caspases are cleaved from an inactive zymogen to an active heterodimer. These active caspases degrade several components critical for cell survival, such as DNA repair elements, structural proteins, and cell signaling peptides [35]. Caspase-3, the primary death effector, serves as a key target for monitoring apoptosis, for it is activated through both cell surface death receptor and mitochondrial release of cytochrome c [36]. Disruption in signaling pathways that regulate apoptosis can lead to a variety of pathologic conditions, making apoptosis an area of intense focus for research [37].

Eight genes related with apoptosis are found in the lower part of Figure 1B. Only two genes showed differential expression in nearly all tumors, *Bik *gene was overexpressed in 14/15 samples and *MCL-1 *gene expression was diminished or absent in the same number of tumors. The behavior of the remaining genes was not constant; in tumor 5, for example, nearly all selected genes were overexpressed, whereas in tumor 6 expression of nearly all selected genes was lost. The heterogeneous behavior of these genes makes it difficult to establish their role in apoptosis or survival of neoplasic cells. However, it was possible to establish which genes, as in the case of the *Bik *gene, are probably related with cancer progression or are involved in other genetic networks.

BH3-only proteins are structurally distant members of the Bcl-2 protein family which triggers apoptosis. These proteins share with each other and with the remainder of the Bcl-2 family only a nine amino acid BH3 (Bcl-2 Homology) region. This domain is required for its ability to bind to Bcl-2-like, pro-survival proteins and to initiate apoptosis. Mammals have at least 10 BH3-only genes that differ in expression pattern and model of activation (regulated by a diverse range of transcription factors). Certain BH3-only proteins, including *Bad, Bik/Nbk, Bid, Bim/Bod, and Bmf*, are restrained by post-translational modifications that cause their sequestration from pro-survival Bcl-2 family members [38]. *Nbk/Bik *(natural-born killer/Bcl-2-interacting killer) is a tissue-specific BH3-only protein which molecular function is largely unknown. *Nbk *fails to induce apoptosis in the absence of *Bax*. *Nbk *interacts with *Bcl-xL *and *Bcl-2 *but not with *Bax*. It was suggested that *Nbk *acts as an indirect killer which triggers Bax-dependent apoptosis, whereas *Bak *is not sufficient to confer sensitivity to *Nbk *[39].

BH3-only proteins require multidomain pro-apoptotic members *Bax *and *Bak *to release cytochrome c from mitochondria and kill cells. Short peptides representing alpha-helical BH3 domains of *Bid *or *Bim *are capable of inducing oligomerization of *Bak *and *Bax *to release cytochrome c. BH3 peptides from *Bad *and *Bik *cannot directly activate *Bax *or *Bak*, but instead bind anti-apoptotic members of BCL-2, resulting in displacement of Bid-like BH3 domains which initiate mitochondrial dysfunction. These data support two types of BH3 domains: Bid-like domains which activate *Bax*, *Bak*, and *Bad-like *domains which sensitize by occupying the pocket of anti-apoptotic members [40].

Bik, a BH3-alone protein (Bcl-2 homology domain), is a pro-apoptotic member of the BCL2 family. Missense *Bik *gene mutations and sequence alterations in intronic regions were observed in cell lymphomas; these data indicate that mutation of the *Bik *gene is relatively frequent [41,42]. The importance of phosphorylation and desphosphorylation reactions in intracellular signaling pathways has long been accepted. The importance of serine/threonine protein phosphatases in many processes including apoptosis is recognized. The phosphorylation state of anti-apoptotic (Bcl-2 and Bcl-xL) and pro-apoptotic (Bad, Bid and Bik) Bcl-2 proteins regulates their cellular activity and, therefore, cell survival and cell death. For instance, desphosphorylation of Bad allows it to interact with Bcl-xL and initiate cell death [43]. However, mutation of the Bik phosphorylation sites, in which the Thr and Ser residues were changed to alanine residues, reduced the apoptotic activity of Bik protein without significantly affecting its ability to heterodimerize with BCL-2 [44]

Of note, a significant fraction of either ectopic or endogenous BIK was found associated with the endoplasmic reticulum, suggesting that this organelle, in addition to mitochondria, may be a target of BIK function [45]. Collectively, the results identify BIK as an initiator of cytochrome c release from mitochondria operating from a location at the ER [46]. These results indicate that any function of Bik in programmed cell death and stress-induced apoptosis must overlap that of other BH3-only proteins [47].

Because the product of *Bik *gene is pro-apoptotic, this protein has been used to induce apoptosis in several cancer cell lines (PC-3, HT-29, MCF-7, MDA-MB-231, 435, 468 and A540) [27,28,48]. Fay M *et al; *in 2003 [49] reported underexpression of the *CUL-5 *gene in breast tumor tissue, whereas expression levels in several cancer cell lines (MCF7, MDA-MB-231) are essentially identical to those of normal breast. Probably breast tumor tissues (i.e., *Bik *gene) have a different behavior *vs*. breast cancer cell lines at a molecular level. Moreover we found some reports with different expression levels of *Bik *gene among multiple human tissues: Daniel T *et al; *1999 [48] reported absence of *Bik *expression in heart, skeletal muscle, and brain, while Verma *et al; *2000 [26] reported higher expression in heart and skeletal muscle. It is difficult to reconcile these reports.

## Conclusion

It is not known whether *Bik *gene works as an apoptosis activator or only sensitizes the cell for death. In any event, it could be a prognostic factor and a possible therapy target in breast cancer. The findings reported in this paper deserve further investigation.

## List of abbreviations

*ATM *Ataxia telangiectasia

*BAD *BCL2-antagonist of cell death

*BAK *BCL2-antagonist/killer 1

*BAX *BCL2-associated × protein

*BCGF1 *B-cell growth factor

*BCL2- A1 *BCL2-related protein A1

*BCL2 *B-cell CLL/lymphoma 2

*Bcl-xL *BCL2-like 1

*CUL-5 *Cullin 5

*BCLW *BCL2-like 2

*Bik/Nbk *BCL2-interacting killer/ natural-born killer

*BMP8 *Bone morphogenetic protein 8

*CASP10 *Apoptotic cysteine protease MCH4

*CASP8 *Apoptotic cysteine protease MCH5

*CCNG1 *Cyclin G1

*CD30-L *CD30 ligand

*CD-40-L *CD40 ligand

*CDC25A *Cell division cycle 25a

*CDC-6 *CDC6-related protein

*CDK 2 *Cyclin-dependent kinase 2

*CDK 5 *Cell division protein kinase 5

*CDK 7 *Cyclin-dependent kinase 7

*CDK5 activator *Cyclin-dependent kinase 5 activator p35

*COL11A1 *Collagen type xi alpha-1

*COL11A2 *Collagen type xi alpha-2

cpm Counts per million

DCC Tumor suppressor protein DCC

*EGFR *Epidermal growth factor receptor

*ERK4 *Extracellular signal-regulated kinase 4

*ERK5 *Extracellular signal-regulated kinase 5

*FGF 3 *Fibroblast growth factor-3

*FGF 5 *Fibroblast growth factor-5

*HPRT *Hypoxanthine-guanine phosphoribosyltransferase

*IFN-alpha *Interferon alpha-c leukocyte

*IFN-beta *Interferon beta-1

*IFN-gamma *Interferon gamma

*IFNGR2 *Interferon gamma accessory factor-1

*IL-13 *Interleukin-13

*IL-3 *Interleukin-3

*IL-4 *Interleukin-4

*IL-9 *Interleukin-9

*IP-10 *IFN-gamma-inducible chemokine IP-10

*ITGAE *Integrin alphae

*JUP *Junction plakoglobin

*K2P *Keratin, type ii cytoskeletal 2 oral

*MCL1 *Myeloid cell leukemia sequence 1

*MIF *Macrophage migration inhibitory factor

*MIF *Macrophage migration inhibitory factor

*MIG *Gamma interferon induced monokine

*MMP10 *Matrix metalloproteinase 10

*MMP12 *Matrix metalloproteinase 12

*MMP13 *Matrix metalloproteinase 13

*MMP3 *Matrix metalloproteinase 3

*MMP8 *Matrix metalloproteinase 8

*MSH2 *DNA mismatch repair protein MSH2

*MSH6 *DNA mismatch repair protein MSH6

NB Normal breast

*NCK5AI *Cyclin-dependent kinase 5 activator isoform p39i

*N-MYC *N-MYC proto-oncogene

*OBCAM *Opioid binding cell adhesion molecule

*p53 *cellular tumor antigen p53

*PCNA *Proliferating cell nuclear antigen

*PGS2 *Dermatan sulfate proteoglycan core protein

*ras-like small *Ras-like small GTPase TTF

*RB1 *Retinoblastoma susceptibility

*RPSA *Laminin 37kd receptor

*TDGF1 *Teratocarcinoma-derived growth factor

*TEK *TYROSINE-PROTEIN KINASE RECEPTOR

TNF Tumor necrosis factor

BH3 Bcl-2 Homology

*Bid *BH3 interacting domain death agonist

*Bim/Bod *Bcl-2 like11 (apoptosis facilitator)

*Bmf *Bcl-2 modifying factor

## Competing interests

The author(s) declare that they have no competing interests.

## Authors' contributions

NG performed microarrays and real-time RT-PCR assays, contributed toward the design of the study and drafted the manuscript. FS participated in the conception and design of the study. HA performed real-time RT-PCR and immunohistochemistry assays. ECQ participated in the conception and design study. IA obtained and performed histopathologic diagnosis of the samples. RP participated in the conception of the study. DA drafted the manuscript, participated in the conception and design of study and its coordination. All authors read and approved the final manuscript.

## Pre-publication history

The pre-publication history for this paper can be accessed here:
